# Anaerobic hydrocarbon biodegradation by alkylotrophic methanogens in deep oil reservoirs

**DOI:** 10.1093/ismejo/wrae152

**Published:** 2024-07-31

**Authors:** Cui-Jing Zhang, Zhuo Zhou, Guihong Cha, Ling Li, Lin Fu, Lai-Yan Liu, Lu Yang, Gunter Wegener, Lei Cheng, Meng Li

**Affiliations:** Archaeal Biology Center, Institute for Advanced Study, Shenzhen University, 518060, Shenzhen, China; Shenzhen Key Laboratory of Marine Microbiome Engineering, Key laboratory of Marine Microbiome Engineering of Guangdong Higher Education Institutes, Institute for Advanced Study, Shenzhen University, 518060, Shenzhen, China; Synthetic Biology Research Center, Institute for Advanced Study, Shenzhen University, 518060, Shenzhen, China; Key Laboratory of Development and Application of Rural Renewable Energy, Biogas Institute of Ministry of Agriculture and Rural Affairs, 610041, Chengdu, China; Key Laboratory of Development and Application of Rural Renewable Energy, Biogas Institute of Ministry of Agriculture and Rural Affairs, 610041, Chengdu, China; Key Laboratory of Development and Application of Rural Renewable Energy, Biogas Institute of Ministry of Agriculture and Rural Affairs, 610041, Chengdu, China; Key Laboratory of Development and Application of Rural Renewable Energy, Biogas Institute of Ministry of Agriculture and Rural Affairs, 610041, Chengdu, China; Key Laboratory of Development and Application of Rural Renewable Energy, Biogas Institute of Ministry of Agriculture and Rural Affairs, 610041, Chengdu, China; Key Laboratory of Development and Application of Rural Renewable Energy, Biogas Institute of Ministry of Agriculture and Rural Affairs, 610041, Chengdu, China; MARUM, Center for Marine Environmental Sciences, University of Bremen, 28359, Bremen, Germany; Max Planck Institute for Marine Microbiology, 28359, Bremen, Germany; Key Laboratory of Development and Application of Rural Renewable Energy, Biogas Institute of Ministry of Agriculture and Rural Affairs, 610041, Chengdu, China; Archaeal Biology Center, Institute for Advanced Study, Shenzhen University, 518060, Shenzhen, China; Shenzhen Key Laboratory of Marine Microbiome Engineering, Key laboratory of Marine Microbiome Engineering of Guangdong Higher Education Institutes, Institute for Advanced Study, Shenzhen University, 518060, Shenzhen, China; Synthetic Biology Research Center, Institute for Advanced Study, Shenzhen University, 518060, Shenzhen, China

**Keywords:** methanogenic hydrocarbon biodegradation, oil reservoirs, Candidatus Methanoliparum, alkylotrophic methanogens, metagenomic

## Abstract

In subsurface biodegraded oil reservoirs, methanogenic biodegradation of crude oil is a common process. This process was previously assigned to the syntrophy of hydrocarbon-degrading bacteria and methanogenic archaea. Recent studies showed that archaea of the *Candidatus Methanoliparum* named as alkylotrophic methanogens couple hydrocarbon degradation and methane production in a single archaeon. To assess the geochemical role of *Ca. Methanoliparum*, we analyzed the chemical and microbial composition and metabolites of 209 samples from 15 subsurface oil reservoirs across China. Gas chromatography–mass spectrometry analysis revealed that 92% of the tested samples were substantially degraded. Molecular analysis showed that 85% of the tested samples contained *Ca. Methanoliparum*, and 52% of the tested samples harbored multiple alkyl-coenzyme M derivatives, the intercellular metabolites of alkylotrophic archaea. According to metagenomic and metatranscriptomic analyses, *Ca. Methanoliparum* dominates hydrocarbon degradation in biodegraded samples from the Changqing, Jiangsu, and Shengli (SL) oilfields, and it is persistently present as shown in a 15-year-long sampling effort at the Shengli oilfield. Together, these findings demonstrate that *Ca. Methanoliparum* is a widely distributed oil degrader in reservoirs of China, suggesting that alkylotrophic methanogenesis by archaea plays a key role in the alteration of oil reservoirs, thereby expanding our understanding of biogeochemical process in the deep biosphere.

## Introduction

Petroleum (crude oil) is a complex mixture consisting of saturated hydrocarbons (including alkanes and cycloalkanes), unsaturated hydrocarbons (including aromatic hydrocarbons), and non-hydrocarbons (including compounds containing carbon, hydrogen, oxygen, sulfur, nitrogen, and other elements). Most reservoir oils exhibit signs of biodegradation, such as the removal of specific compound classes, primarily *n*-alkane derivates with long alkyl chains [1, 2]. Deep subsurface oil reservoirs are anoxic [3], hence it is widely accepted that anaerobic hydrocarbon degradation must have taken place [4]. Microbial anaerobic degradation of petroleum hydrocarbons in oil reservoirs plays a vital role in carbon cycling and leads to the formation of biodegraded oils [5].

Over the past three decades, multiple anaerobic hydrocarbon-degrading bacteria within the phyla *Proteobacteria*, and *Firmicutes* have been cultured [6]. The most characterized anaerobic hydrocarbon degradation metabolism by bacteria is the fumarate addition. Hydrocarbon-degrading bacteria contain alkylsuccinate synthase (ASS/MAS) or benzylalkylsuccinate synthases (BSS), which ligate alkanes to fumarate to form an alkyl-succinate [7–9]. Another anaerobic hydrocarbon degradation is the activation of aromatic alkanes by the ethylbenzene dehydrogenase [10]. Hydrocarbon-degrading bacteria utilize electron acceptors, such as nitrate, iron, and sulfate [6]. In the absence of electron acceptors, some of these bacteria can also grow in syntrophic relationships with methanogens [11–13]. In this case, the bacteria ferment hydrocarbons into simple compounds like acetate, formate, and hydrogen, and methanogens consume these acetate, formate, and hydrogen and use the methyl-coenzyme M reductase (MCR) for methane formation, an enzyme that is only found in archaea [5, 12].

The degradation of hydrocarbons by bacteria has been widely investigated for decades [14]. Only recently, anaerobic hydrocarbon-degradation was found in archaea, implying that anaerobic hydrocarbon degrading microorganisms have a wider lineage than originally suspected. Hydrocarbon-degrading archaea utilize alkyl-coenzyme M reductase (ACR), an enzyme that, apart from MCR, is for the activation of multi-carbon alkanes [15]. Archaea of the GoM-Arc1/*Ca. Argoarchaeum*/*Ca. Ethanoperedens* and *Ca. Syntropharchaeales* use Acr for the growth on ethane and butane [16–18]. The archaeon *Alkanophaga* grows with alkanes ranging from pentane to tetradecane [19]. The archaeon *Cerberiarchaeum* from *Hadarchaeota* can grow with hexadecane [20]. ACR has been identified in several members of the *Archaeoglobales* [21], *Ca. Bathyarchaeota* [22], and *Ca. Helarchaea* [23], suggesting that alkanotrophy might be better than this metabolism is found in many different archaeal lineages [24]. Genomic information and physiological experiments suggest that most of the alkane-oxidizing archaea transfer the excess of reducing equivalents from alkane oxidation to sulfate reducing partner bacteria. Recent studies based on metagenome-assembled genomes (MAGs) suggest that *Ca. Methanoliparia* contains both ACR and MCR, coupling alkane oxidation to methanogenesis within single cells [15, 25]. A combination of cultivation, metagenomics, and metabolite analysis confirmed in the laboratory that *Ca. Methanoliparum* activates long-chain alkanes (C_13+_) and long-chain alkyl-substituted hydrocarbons with ACR variants [26]. However, it is unknown whether *Ca. Methanoliparum* distributes widely in natural oil reservoirs.

**Figure 1:**
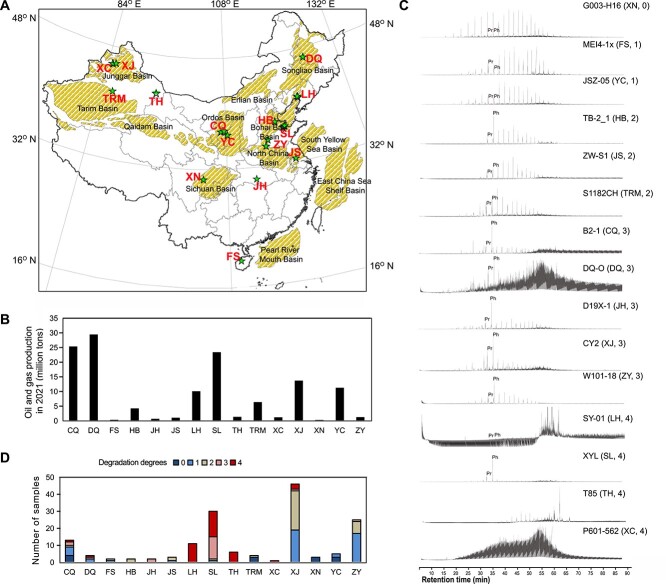
Origin of crude oil samples, and their degradation degree. (A) Geographic map of oil basins (dashed areas), and sampling locations (stars) across China. (B) Bar plot insert depicts the oil, and gas production of the different oil fields. (C) Gas chromatograms of the dichloromethane extracts of selected samples for each oilfield. The names of samples are highlighted in Table S2. Sampling source, and degradation degrees marked by numbers are shown in brackets. Pr: Pristane; Ph: Phytane. (D) Number of samples from each oilfield. The degradation degrees are indicated by the color code. A score of 0 indicates non-biodegradation, characterized by a straight TIC mass spectrometry curve, and the presence of *n*-alkanes. A score of 1 represents light degradation, identified by a slight UCM in the TIC curve, and mild degradation of *n*-alkanes (with C_17_, and C_18_ concentrations higher than Pr, and Ph). Scores of 2, and 3 indicate moderate degradation, characterized by a significant UCM in the TIC curve, and noticeable degradation of *n*-alkanes (with C_17_, and C_18_ concentrations lower than Pr, and Ph, and Pr, and Ph being significantly higher than other *n*-alkanes). A score of 4 indicates severe biodegradation, identified by a significant UCM in the TIC curve, and complete degradation of *n*-alkanes. Abbreviations of oilfields are noted. CQ: Changqing, DQ: Daqing, FS: Fushan, HB: Huabei, JH: Jianghan, JS: Jiangsu, LH: Liaohe, SL: Shengli, TH: Tuha, TRM: Tarim, XC: Xinchun, XJ: Xinjiang, XN: Xinan, YC: Yanchang, ZY: Zhongyuan.

In this study, we collected 209 samples from 15 oil fields across China (Fig. 1A, Table S1) and analyzed the abundance and activity of *in-situ* alkylotrophic methanogens by applying multiple methods, including qPCR, 16S rRNA gene amplicon sequencing, metagenomic sequencing, metatranscriptomic sequencing, and alkyl-CoMs detection. The results from this study provide deep insights for the ecological roles of *Ca. Methanoliparum* in oil reservoirs.

## Materials and methods

### Site information and sampling strategy

A total of 209 samples were collected from 15 oilfields representative for distinct geographic distribution across China, including Changqing (CQ), Daqing (DQ), Fushan (FS), Huabei (HB), Jianghan (JH), Jiangsu (JS), Liaohe (LH), Shengli (SL), Tuha (TH), Tarim (TRM), Xinchun (XC), Xinjiang (XJ), Xinan (XN), Yanchang (YC), and Zhongyuan (ZY) (Fig. 1A). The samples were grouped into four types, including crude oil, oily sludge, oil production water, and hydrocarbon source rock. Oily sludge comes from the sand and gravel brought out during the extraction of crude oil, which is obtained by extracting the upper crude oil after static settlement. The produced water comes from the water drive cycle treatment station. These oilfields are located in the major hydrocarbon basins of both marine and terrestrial origins in China. Among them, seven oilfields produced more than 5 million tons of crude oil and gas in 2021 [27] (Fig. 1A and B). The map (Fig. 1A) was drawn by ArcGIS v10.5 (http://www.esri.com/arcgis/about-arcgis). The sampling campaign for this study included several trips from 2007 to 2022. The samples come from different depths ranging from 954 to 5648 m. Details on sample types, sampling site location, sampling date, sampling depth, and the storage of samples can be found in Table S1. Samples collected continuously from 2007 to 2022 from the SL oilfield were stored in −80°C refrigerator (MDF-330, SANYO, Japan). Samples from other oilfields were stored anoxic in gas-tight bags at 4°C in a refrigerator (SC-328DS, Haier, China) for subsequent microbial enrichment experiments. The oil sludge samples were sealed in plastic bags (Separation, China). The gas-tight bags were made of plastic, with dimensions of 70^*^100 cm (length^*^width). The bags were sealed by using the zipper structure at one end to avoid oxygen penetration. The collected water and crude oil were stored in plastic containers (Wangxiang plastics, China) with capacities of 10 L and 550 ml, respectively.

### Extraction of crude oil, GC–MS analysis, and identification of crude oil degradation levels

We extracted crude oil using dichloromethane from a total of 157 samples (Table S2). Approximately 0.5 g of oil or oily sludge were transferred into a 10 ml glass vials, and 5 ml dichloromethane (Kelong) was added. After letting stand for 24 h, 1.5 ml of the DCM phase was carefully transferred into 2 ml sample vial for GC–MS analysis. Hydrocarbon source rock was dried first, then powdered to <60 mesh using an agate mortar (Fritsch Pulverisette, Germany). About 2.5 g of homogenized powder rock sample was placed in the thimble and placed in the Soxhlet apparatus. Then the apparatus was fitted to a distillation flask, which was filled with 250 ml of dichloromethane. During the Soxhlet extraction, the flask was heated to 50°C–60°C. After extraction, samples with dichloromethane were air dried at room temperature until the final volume was around 10 ml. About 1.5 ml of sample was transferred into a 2 ml sample vial for GC–MS analysis.

Dichloromethane extracts were analyzed by gas chromatography coupled to mass spectrometry using an Agilent 7890A GC with mass-selective detector, equipped with a 30 m × 0.32 mm HP-5 column with a film thickness of 0.25 μm (Agilent), and a flow rate of 1.2 ml min^−1^. The GC oven temperature was initially held at 50°C for 3 min, then ramped to 70°C at 5°C/min, then ramped to 280°C at 4.5°C/min. The mass spectrometer was operated in full scan mode.

Degradation of crude oil was evaluated on a scale ranging from 0 to 4. This scale was adapted from the modified P-M degradation method [28] described previously [29]. A score of 0 indicates non-biodegradation, characterized by a straight Total Ion Chromatogram (TIC) mass spectrometry curve, and the presence of *n*-alkanes. A score of 1 represents light degradation, identified by a slight Unresolved Complex Mixture (UCM) in the TIC curve, and mild degradation of *n*-alkanes (with C_17_ and C_18_ concentrations higher than Pristane and Phytane). Scores of 2 and 3 indicate moderate degradation characterized by a significant UCM in the TIC curve, and noticeable degradation of *n*-alkanes (with C_17_ and C_18_ concentrations lower than Pristane and Phytane, and Pristane and Phytane being significantly higher than other *n*-alkanes). A score of 4 indicates severe biodegradation, identified by a significant UCM in the TIC curve, and complete degradation of *n*-alkanes.

### DNA extraction, amplification, sequencing, and quantification of 16S rRNA genes

DNA was extracted using 0.5 g of each oil, oily sludge, or hydrocarbon source rock by a bead-beating as previously described [30]. For oil production water, 500 ml of produced water was passed through a 0.22 μm filter, and the filer was extracted as described above. Archaeal community structures were analyzed by archaeal 16S rRNA gene amplicon sequencing with the primer pairs Arch519F (5′-CAGCCGCCGCGGTAA-3′), and Arch915R (5′-GTGCTC CCCCGCCAATTCCT-3′) [31]. Amplifications were performed with 34 thermal cycles of 94°C for 1 min, 57°C for 45 s, and 72°C for 1 min. Amplicon libraries required at least 150 ng of PCR products for sequencing on the NovaSeq 6000 platform with the PE250 strategy. A total of 138 amplicon sequencing libraries were generated using a NovaSeq 6000 System (Illumina) with paired-end 250 bp mode (PE250) at Novogene Bioinformatics Technology Co., Ltd (Tianjin, China) (Table S3). Raw reads were filtered as previously described [32, 33]. Quality-filtered reads were loaded into the QIIME2 pipeline [34]. Feature tables and feature sequences were generated using DADA2. Amplicon sequence variants (ASVs) were classified using q2-feature-classifier’s *classify-sklearn* method and were then taxonomically assigned by using the Naive Bayes method implemented in QIIME2, with the SILVA NR99 database (release 138) as [35]. *Ca. Methanoliparum* was not recognized by the QIIME2 classification. The full-length 16S rRNA gene sequences of *Ca. Methanoliparum* obtained from our previous study, and public database were used as the representative sequences (Supplementary Material File 1). We performed a sequence similarity search against the representative sequences database using BLASTn, and four feature sequences corresponding to four clusters of *Ca. Methanoliparum* were retained with identity = 100% and coverage = 100%. To calculate the relative abundance of *Ca. Methanoliparum*, we used the number of reads for these four features divided by the total number of reads in each sample.

The abundance of *Ca. Methanoliparum* in samples was assessed by quantitative real-time PCR with the primer set Mlp1F/Mlp1R (5′-GGGAATTCGACTAAGCCATGCAA-3′/5′-CCCGGCCCTTTCTATTAGGTG-3′) [26]. Triplicate amplifications were conducted in a 10 μl reaction system containing 5 μl of SsoFast EvaGreen Supermix (Bio-Rad), 3.25 μl of sterilized distilled H_2_O, 0.25 μl of each primer (10 μM), 0.25 μl of bovine serum albumin (5 mg ml^−1^), and 1 μl of template DNA (~10 ng μl^−1^). The thermal cycling steps were performed using the qPCR instrument (Bio-Rad CFX96), which consisted of an initial denaturation step at 95°C for 3 min, 45 cycles of 95°C for 15 s, 65°C for 15 s, and 72°C for 10 s with plate reading, and a final extension step at 72°C for 10 min. After the main program, melt curve analysis was performed from 65°C to 95°C, with an increment of 0.5°C and 0.5 s plate reading at each step. The sample DNA was diluted for ~10 to 100 times when necessary. The standard curve was generated with tenfold serially diluted linear plasmids containing a single copy of *Ca. Methanoliparum* 16S rRNA gene.

### Metagenome sequencing, assembly, genome binning, and annotation

To characterize the metabolic potentials of *Ca. Methanoliparum*, we selected the most degraded samples from every oilfield for metagenomic sequencing. DNA was extracted in the same way as above. We used the Agilent 5400 Fragment Analyzer System (Agilent Technologies, USA) to analyze the size and integrity of the DNA, and Nanodrop 2000 (Thermo Scientific, USA) to check the purity of the DNA. Once the DNA samples passed the quality check, we used a Covaris S220 ultrasonic disruptor (Gene Company Ltd, USA) to randomly fragment the DNA. Subsequently, we completed the entire library preparation process, including end repair, A-tailing, adapter ligation, purification, and PCR amplification. After the library construction was complete, we initially quantified it using Qubit 2.0 (Thermo Scientific, USA) and then diluted the library. We then used Agilent 2100 (Agilent Technologies, USA) to detect the insert fragment size of the library. Once the insert fragment size met expectations, we used qPCR to accurately quantify the effective concentration of the library to ensure its quality. After passing library quality control, we pooled different libraries to the flow cell based on their effective concentration and the desired amount of target data for sequencing. The libraries were then clustered by cBOT and sequenced using the NovaSeq 6000 System. We used 50 ng of DNA for sequencing with the PE150 mode for each metagenomic sample. A total of 28 metagenomic sequencing libraries were prepared by Novogene with their in-house pipelines (Table S4). More than 15 Gbp of raw sequence data were obtained for each sample. The raw reads were quality trimmed using Trimmomatic with default parameters [36] and assembled using metaSPAdes with the following parameters: -k 21, 33, 55, 77, 99, 127 [37]. The assembled fragments were binned into MAGs using MetaWRAP (integrating MetaBAT2, CONCOT, and MaxBin2 methods) with the default parameters [38]. The quality of MAGs was evaluated using CheckM [39]. Taxonomic classification of MAGs was performed against the Genome Taxonomy Database with GTDB-Tk (R207 v2) [40]. We obtained 2196 medium-to-high quality MAGs (completeness >70% and contamination <10%) [41]. The MAGs were translated by Prodigal using the “-p meta” parameters [42]. For each predicted coding sequence (CDS), protein function was annotated automatically using the Kyoto Encyclopedia of Genes, and Genomes (KEGG) server [43], and eggNOG-mapper [44]. All the MAGs from 28 metagenomic datasets were dereplicated using dRep [45] with an ANI cut-off of 97%. After dereplication, a total of 1346 medium-to-high quality MAGs were retained (Table S5).

### Identification of *acrA* and *assA*/*bssA*/*nmsA* genes

To identify *acrA* genes involved in the anaerobic degradation of hydrocarbons by archaea, HMM profiles for the alkyl-CoM reductase alpha subunit (PF02249 for MCR_alpha and PF02745 for MCR_alpha_N) from the PFAM database were used. To identify *assA*/*bssA*/*nmsA* genes involved in the anaerobic degradation of hydrocarbons by bacteria, a custom hidden Markov model (HMM) profile (Supplementary Material File 2) was constructed based on the protein sequences of the alpha subunits from alkyl-succinate synthase/(1-methylalkyl) succinate synthase (AssA/MasD), benzylsuccinate synthase (BssA), and naphtylmethylsuccinate synthase (NmsA) (Supplementary Material File 3). Scaffolds with length > 500 bp from 28 metagenomic assemblies were kept, and coding sequences were predicted with Prodigal (v.2.6.3) using the “-p meta” parameter [42]. Putative marker genes were screened using the profiles described above with hmmsearch (HMMER v.3.1b2) [46] and validated by phylogenetic analysis (as described below). The *acrA* and *assA*/*bssA*/*nmsA* genes were dereplicated with 99% nucleic acid identity using CD-hit [47]. Nine *acrA* and 80 *assA*/*bssA*/*nmsA* genes (Supplementary Material File 4) were retained. These sequences were used as references for metatranscriptomic reads mapping.

### Phylogenetic analyses of *Ca. Methanoliparum* MAGs and functional genes

A phylogenetic tree was constructed based on the concatenation of 16 ribosomal proteins (L2, L3, L4, L5, L6, L14, L15, L16, L18, L22, L24, S3, S8, S10, S17, and S19) [41]. The predicted protein sequences for each MAG were searched with Prodigal in CheckM [39]. All proteins were aligned with MUSCLE [48] and trimmed with trimAL [49]. A maximum-likelihood tree was inferred using IQ-Tree [50] (setting: -m WAG -bb 1000 -alrt 1000) based on the concatenation of trimmed 16 ribosomal proteins. The trees were visualized using the Interactive Tree of Life (iTOL) [51].

For the phylogenetic analysis of AcrA/McrA, reference sequences were retrieved from fungene (http://fungene.cme.msu.edu/) and recent publications [52]. For the phylogenetic analysis of AssA-related, sequences used for the AssA custom HMM profile construction (Supplementary Material File 4) were used as references. The amino acid sequences of AcrA/AssA-related were retrieved from metagenomic assembled fragments using hmmsearch (HMMER v.3.1b2) [46] as described above. The amino acid sequences of AcrA/McrA and AssA related proteins were aligned using MUSCLE [48], respectively. Phylogenetic trees for AcrA/McrA and AssA-related protein sequences were constructed using FastTree 2 [53] (Supplementary Material Files 5 and 6). All trees were visualized in iTOL [51].

### Metatranscriptomics workflow and evaluation of the activity of *Ca. Methanoliparum*

Total RNA was isolated from 36 selected samples using an acid-phenol chloroform isoamyl alcohol-based protocol as described before [54]. The detailed information is listed in Table S6. We used Nanodrop 2000 (Thermo Scientific, USA) to determine the nucleic acid concentration and the Agilent 5400 Fragment Analyzer System (Agilent Technologies, USA) to assess the integrity and purity of the nucleic acids, after obtaining total nucleic acids. Next, we used two reagent kits to eliminate DNA and rRNA and enrich mRNA (TianGen kit, Beijing). The mRNA was then fragmented using a fragmentation buffer, and single-stranded cDNA was synthesized using a six-base random primer with mRNA as a template. Subsequently, buffer, dNTPs (with dTTP replaced by dUTP), DNA Polymerase I (TianGen, Beijing), and RNase H (TianGen, Beijing) were added to synthesize double-stranded cDNA. The double-stranded cDNA was then purified using AMPure XP beads, followed by degradation of the second strand of cDNA containing U using the USER enzyme. The purified double-stranded cDNA underwent end repair, A-tailing, and adapter ligation, and size selection was performed using AMPure XP beads (Beckman, USA). PCR amplification was then conducted, and the PCR products were purified using AMPure XP beads to obtain the final library. After constructing the library, we initially quantified it using Qubit2.0 Fluorometer, diluting the library to 1.5 ng/μl. Subsequently, we used an Agilent 2100 bioanalyzer to detect the insert size of the library. Once the insert size met expectations, qRT-PCR was used for accurate quantification of the library’s effective concentration (with an effective concentration above 2 nM) to ensure library quality. After passing library quality control, different libraries were pooled based on effective concentration and the desired amount of target data for Illumina sequencing. Metatranscriptomic sample library construction required 100 ng of cDNA for sequencing on the NovaSeq 6000 platform with the PE150 strategy.

To estimate the activity of *Ca. Methanoliparum*, and hydrocarbon-degrading bacteria, the abundance of transcripts was calculated at both the genome and gene level. At the genome level, the abundance of 1346 dereplicated MAGs was determined by mapping the metatranscriptomic reads to the contigs of the MAGs using Bowtie2 [55]. The resulting SAM mapping files were converted to BAM files using SAMtools [56]. The read coverage in MAG contigs was calculated using BEDTools [57]. The number of reads was normalized to the length of MAGs. The relative abundance of each MAG is the number of normalized reads mapped to individual MAGs divided by the total number of normalized metagenomic reads mapped to all dereplicated MAGs (Table S7).

The transcription activity of *acrA* genes of *Ca. Methanoliparum*, and *assA*-related genes of hydrocarbon-degrading bacteria was calculated by mapping the metatranscriptomic reads to the annotated genes using Burrows–Wheeler Aligner (BWA) with the default settings [58]. The resulting SAM mapping files were converted to BAM files using SAMtools. The read coverage in MAG contigs was calculated using BEDTools. We used the fragments per kilobase of transcript per million mapped reads (FPKM) values to normalize the expression level (Table S8).

### Global distribution of *Ca. Methanoliparum* in oil fields

To reveal the global distribution of *Ca. Methanoliparum* in other oilfields, we acquired gene sequence data by three independent targeted approaches. First, all 16S rRNA gene sequences longer than 400 bp that exhibited >97% sequence similarity relative to *Ca. Methanoliparum* were screened against the April 2022 NCBI Sequence Read Archive using the Integrated Microbial Next Generation Sequencing (IMNGS) [59]. Second, all 16S rRNA gene sequences that exhibited >97% similarity relative to *Ca. Methanoliparum* were retrieved against the NCBI NR database using BlastN. Third, we searched Web of Science (December 2022) with the keyword combination “oilfield,” and “microorganism,” and “16S rRNA sequencing”. We retrieved 214 references based on the criteria: (i) they contained oil field samples and (ii) they provided accession numbers for 16S rRNA gene sequences in NCBI. A BlastN search against 16S rRNA gene sequences of *Ca. Methanoliparum* retrieved sample sites that contained *Ca. Methanoliparum*. Results obtained from the combination of all the above three independent approaches are listed in Table S9.

### Metabolite extraction and mass spectrometry analysis

We extracted metabolites from a total of 166 samples (Table S10). A volume of 0.3 ml or 0.3 g of the sample was placed in a 2 ml microtubes (SARSTEDT) and mixed with 1 ml of acetonitrile: methanol: water (40:40:20, v/v/v) and 0.3 g of sterile glass beads (diameter 0.1 mm; Sigma-Aldrich). The cells were lysed by oscillating the homogenizer (FastPrep-24, MP) at 6 m/s for 50 s three times. The mixture was then centrifuged to remove the glass beads and cellular debris (12 000 *g*, for 10 min at 4°C). The cell extract, which was prepared by adding it to 10 ml of deionized water, was analyzed by high-performance liquid chromatography (LC-30AD, Shimadzu) coupled with mass spectrometry on AB SCIEX 4500. The chromatography was performed with a reversed-phase C18 column (1.7 μm, 2.1 × 150 mm, 75 cm, Thermo), operated at a constant 30°C flow rate of 0.3 ml/min. The mobile phase A was ultrapure water with 0.5% ammonium acetate (Sigma-Aldrich) and the mobile phase B was acetonitrile (Sigma-Aldrich). The gradient was 0–3.0 min, 15%–90% B; 3.0–5 min, 90% B; 5.0–5.1 min, 90%–15% B; 5.1–8.0 min, 15% B. For each run, the injection volume was 10 μl. In ionization, the curtain gas was nitrogen with a pressure of 30 psi, and a temperature of 450°C; the ion source gas 1, and gas 2 was 50 psi; the scan rate was 200 Da/s; the collision energy was 55 V; and the ionspray voltage was 4.5 kV. Based on the measurement of authentic hexadecyl-CoM, and eicosyl-CoM, standards, the alkyl-CoMs with a generalized formula of C_n_H_2n + 1_S-C_2_H_4_SO_3_^−^ will fragment into HSO_3_^−^, m/z = 80.9; C_2_H_3_SO_3_^−^, m/z = 106.9; C_n_H_2n + 1_SO_3_^−^, m/z ≈ 12.00055 ^*^  *n* + 1.00837 ^*^ (2*n* + 1) + 79.9 with a machine accuracy of one decimal place. The multiple-reaction monitoring mode (MRM) was used to detect various alkyl-CoM compounds. An ion pair intensity of alkyl-CoM/HSO_3_^−^ >2000 was considered as threshold for the detection of the respective alkyl-CoM.

### Ridge regression of gene expression data

In order to determine the quantitative relationship between the microorganisms (archaea encoding *acrA* or bacteria encoding *assA*/*assA*-like genes), and the degree of crude oil degradation, ridge regression model analysis is carried out [60]. The normalizing function is the natural logarithm **X***_i_* = log for FPKM values of genes based on metatranscriptomic data (see Table S8), and **Y***_i_* = logit for hydrocarbon degradation degree (see Table S2). Consider the setting where observed data are realizations of {(**X***_i_*,**Y***_i_*)}^n^_*i* = 1_ with *p*-dimensional covariates **X***_i_*∈(0,1), and univariate continuous response variables **Y***i*∈{0,1,2,3,4}. A simple regression model has formed.


$$ {\mathbf{Y}}_i={{\mathbf{X}}_i}^{\top}\boldsymbol{\mathrm{\beta}} +{\mathbf{k}}_{\mathrm{i}} $$

where **β** is the vector of regression coefficients, and *k* is the *i*th error component. For simplicity, we assume that the intercept is zero. In case it exists, by centering the observations, one can eliminate it from the study. All computations were conducted using the statistical software R 3.4 to develop the package Rfit for calculating the proposed estimators, their test statistics, and powers described in this section. Ridge regression model was conducted using the software Python 3.7 program. The Python codes are available at GitHub (https://github.com/cui-jing/Ridge-regression-model-python).

## Results

### Degradation degrees across subsurface oil reservoirs in China

A total of 157 oil-related samples listed in Table S2 were analyzed by GC–MS. The chromatograms of the most degraded samples for each oilfield are displayed (Fig. 1C). In total, 92% (144/157) of the tested oil-related samples displayed slight to severe degradation (Table S2). The degradation degree varied across different oilfields (Fig. 1D). All samples from the XN oilfield were non-degraded, whereas all or most samples from other oilfields exhibited degradation. A considerable fraction of samples from Liaohe (LH) (11/11), Shengli (SL) (15/30), and Tuha (TH) (6/6) were severely degraded.

### Abundance and activity of *Ca. Methanoliparum* across subsurface oil reservoirs in China

Among the 2196 medium-to-high quality MAGs from 28 samples (Table S4) from nine oil fields, only MAGs affiliated to *Ca. Methanoliparum* contained Acr (highlighted in Supplementary Material File 6), and there were 38 MAGs classified as *Ca. Methanoliparum* (Table S11, Supplementary Material File 7). Phylogenetic reconstruction of ribosomal protein phylogeny of these MAGs confirmed their affiliation to *Ca. Methanoliparum* (Fig. S1). These MAGs were obtained from the CQ, JS, SL, and XJ oilfields (Table S11). Annotation revealed that 20 of these 38 MAGs contained both Acr and Mcr and encoded core proteins for potential steps in the beta-oxidation pathway, Wood–Ljungdahl pathway, and methanogenesis pathway (Table S12). After dereplication, four clusters of *Ca. Methanoliparum* were retained (Table S5). Fifteen MAGs contained *assA*/*bssA*/*nmsA.* These MAGS belong to 11 microbial taxa, including RBG-13-55-18, BuS5, *Desulfatibacillaceae*, SURF-3, JADFWA01, *Smithellaceae*, UBA4767, JS1, *Atribacterota*, *Pseudopelobacteraceae*, and *Desulfitobacteriaceae*.

**Figure 2:**
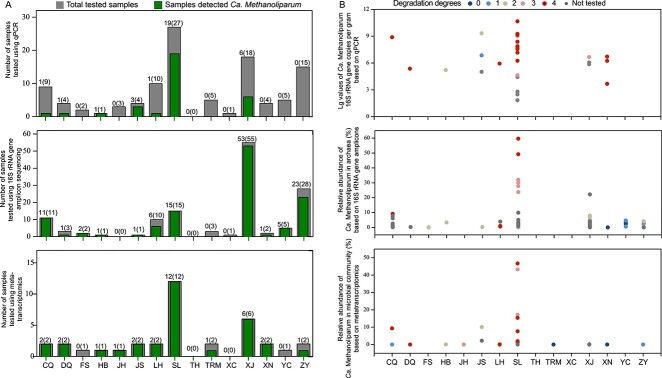
Abundance, and activity of alkylotrophic methanogens across different oil reservoirs, and different degradation degrees. (A) Top panel: Number of samples that were tested using qPCR; middle panel: Number of samples that were tested using 16S rRNA gene amplicon sequencing; bottom panel: Number of samples that were tested by metatranscriptomics. The total number of samples, and the number of samples detected *Ca. Methanoliparum* are numbers in brackets, and numbers out of brackets, respectively. (B) Top panel: Absolute abundance of *Ca. Methanoliparum* based on 16S rRNA gene copies based on qPCR (logarithmic scale, basis 10); middle panel: Relative abundance of *Ca. Methanoliparum* of all archaea based on 16S rRNA gene amplicon sequencing. Bottom panel: Relative abundance of *Ca. Methanoliparum* of all organisms based on mapping of the metatranscriptomes on the entire 1346 dereplicated MAGs (Table S5).

Based on three different molecular analyses (qPCR, 16S rRNA gene amplicon sequencing, and metatranscriptome sequencing), we found that most oil reservoirs contained *Ca. Methanoliparum*, except for TH and XC (Fig. 2A). *Ca. Methanoliparum* 16S rRNA gene quantified by qPCR showed a range of 10^1^–10^10^ gene copies per gram of sample (Fig. 2B, Table S13). The relative abundance of *Ca. Methanoliparum* based on archaeal 16S rRNA gene amplicon sequencing ranged from 0.01% to 59.6% (Fig. 2B, Table S13). The transcripts mapped to *Ca. Methanoliparum* account for 0.01% to 46.7% of the transcriptionally active microbial community (Fig. 2B, Table S13). In total, the abundance and activity of *Ca. Methanoliparum* varied strongly across different oil reservoirs, and different degradation degrees (Fig. 2B). *Ca. Methanoliparum* was most abundant and active in the SL oil reservoirs. Relatively high abundance and transcriptionally active *Ca. Methanoliparum* were also identified in the CQ, DQ, HB, JS, and XJ oilfields. However, *Ca. Methanoliparum* was <1% of archaeal 16S rRNA gene sequences in the FS oilfield, and transcripts of *Ca. Methanoliparum* were <1% of the transcriptionally active microbial community in the JH, and TRM oilfields (Fig. 2B). Overall, *Ca. Methanoliparum* was detected in 85% (147/173) of tested samples using at least one method (qPCR, 16S rRNA gene amplicon sequencing, or metatranscriptome sequencing) (Table S13). Our findings revealed that relative abundance of *acrA*/*Ca. Methanoliparum* increased with degradation degrees (Fig. S2). *Smithellaceae* was the most active hydrocarbon-degrading bacteria in oil reservoirs, showing higher expression in samples with biodegradation degrees between 0 (TRM, XN), 1 (CQ, YC, ZY), 2 (FS, TRM), 3 (DQ, JH), and 4 (DQ, LH, XJ) (Fig. S3).

### Variety of alkyl-CoM derivatives across subsurface oil reservoirs in China

**Figure 3:**
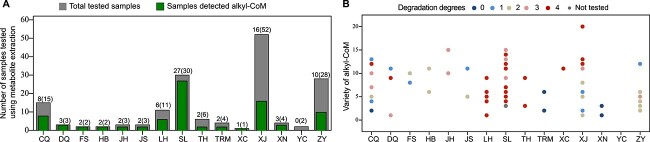
Detection of alkyl-CoM derivatives in different oil reservoir samples. (A) Number of samples across different reservoirs. The total number of samples, and the number of samples detected alkyl-CoM are numbers in brackets, and numbers out of brackets, respectively. (B) Variety of alkyl-CoM derivatives across different reservoirs, and different degrees. Variety is determined according to the carbon number of the alkyl chain.

Alkyl-CoMs are the key initial metabolite in the degradation of short- to long-chain alkanes by *acr*-containing archaea cultures [16, 17, 19, 20, 26]. We detected various alkyl-CoM derivatives (from C_4_H_9_-CoM to C_28_H_57_-CoM) in most oilfields, with an exception for the YC oilfield (Fig. 3A). The variety of alkyl-CoM derivatives ranged from 1 to 20 across different oil reservoirs and different degradation degrees (Fig. 3B). The variety of alkyl-CoM derivatives was most abundant in one oily sludge sample of the XJ oil reservoir, which contained 20 alkyl-CoM derivatives (Fig. 3B, Table S10). In some samples of the CQ, DQ, HB, JH, JS, SL, XC, XJ, and ZY oilfields, more than 10 alkyl-CoM derivatives were detected (Fig. 3B, Table S10). Overall, alkyl-CoM was detected in 52% (86/166) of tested samples (Table S10).

### Long-term observation of *Ca. Methanoliparum* in the SL oilfield

**Figure 4:**
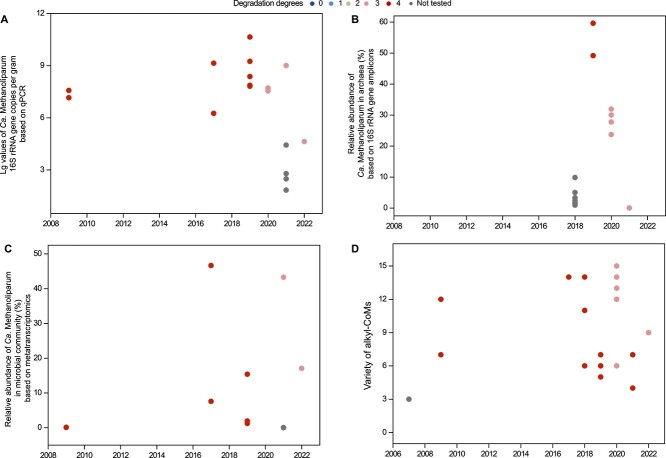
Abundance and activity of *Ca. Methanoliparum* at different times in the SL oilfield. (A) Lg values of *Ca. Methanoliparum* 16S rRNA gene copies based on qPCR. (B) Relative abundance of *Ca. Methanoliparum* in archaea based on 16S rRNA gene amplicon sequencing. (C) Relative activity of *Ca. Methanoliparum* based on mapping the metatranscriptomic reads to all 1346 dereplicated MAGs (Table S5). (D) Variety of alkyl-CoM derivatives in different times in SL oilfields. Variety is determined according to the carbon number of the alkyl chain.

In the SL oilfield, molecular analysis demonstrated consistently high abundance and activity of *Ca. Methanoliparum* over the period from 2007 to 2022 (Fig. 4). The abundances of *Ca. Methanoliparum* exceeded 10^4^ gene copies per gram of sample, except for oil production water samples collected in 2021 (Fig. 4A). The relative abundance of *Ca. Methanoliparum* surpassed 10% of archaeal 16S rRNA gene sequences, except for oil production water samples collected in 2018 and 2021 (Fig. 4B). Metatranscriptomic results show that *Ca. Methanoliparum* accounted for more than 5% of the transcriptionally active microbial communities in oily sludge samples from 2017 to 2022 (Fig. 4C). In addition, we detected a high abundance of alkyl-CoM derivatives in the long time-series samples collected from 2007 to 2022 (Fig. 4D). Overall, detection of *Ca. Methanoliparum* and alkyl-CoM supported that alkylotrophic methanogenesis was persistently present in the SL oilfield in the long term.

## Discussion

### Distribution and diversity of *Ca. Methanoliparum* in oil reservoirs

We screened all environmental samples for the co-occurrence of *Ca. Methanoliparum* 16S rRNA gene sequences based on qPCR, *Ca. Methanoliparum acrA* expression based on metatranscriptomic analysis, and alkyl-CoMs based on metabolites analysis as markers for the presence of alkylotrophic methanogenesis and found four characteristic groups (Table S14). In the first group of samples, 16S rRNA gene sequences of *Ca. Methanoliparum* co-occurred with *acrA* transcripts and various alkyl-CoM derivatives. Such samples all belonged to oily sludge samples with biodegradation degrees between 2 (HB, JS), 3 (SL, XJ), and 4 (CQ, SL). We consider these samples as clearly positive for alkylotrophic methanogenesis. In the second group, 16S rRNA gene sequences of *Ca. Methanoliparum*, and alkyl-coenzyme M were present, but the *acrA* was not expressed. Such samples all belonged to oily sludge samples with biodegradation degrees 4 (DQ, LH, and XJ). RNA is highly labile and might not have survived the recovery and extraction of samples. In the third group, alkyl-coenzyme M was present, but neither 16S rRNA gene sequences of *Ca. Methanoliparum* nor *acrA* transcripts were detected. It might well be that alkyl-CoMs preserve better in those samples than DNA/RNA, suggesting alkyl-CoMs is a potential biomarker candidate for alkylotrophic methanogenesis in oil reservoir environments. According to the degradation degree of 0–4, the proportions of alkyl coenzyme M that can be detected in the samples are 64%, 33%, 50%, 100%, and 78% (Table S10). In the samples with a degradation degree of 3, the probability for the detection of alkyl-coenzyme M variants is close to 100%. In samples of the degradation degree 4 neither 16S rRNA gene sequences of *Ca. Methanoliparum*, noralkyl-coenzyme M, nor *acrA* expression were detected. Reasons for this might be the total depletion of substrates (i.e. in completely biodegraded samples LH), the degradation of RNA during the storage of the samples, or other inhibitory factors, such as temperature changes. These samples were rare, suggesting alkylotrophic methanogenesis is widely distributed in most Chinese oil reservoirs.

To assess their distribution in oil reservoirs worldwide, we also collected the 16S rRNA gene sequences of *Ca. Methanoliparum*, and their habitat information (Fig. S4, Table S9). *Ca. Methanoliparum* appeared in 10 different oilfields, including those in Australia, Canada, Costa Rica, India, Japan, and the USA, suggesting a global distribution of *Ca. Methanoliparum* in hydrocarbon-rich habitats. *Ca. Methanoliparum* appears in the Niibori oilfield in Japan, which has never experienced water flooding, suggesting that *Ca. Methanoliparum* is likely indigenous to the oil reservoirs and not introduced via drilling or water flooding [61]. *Ca. Methanoliparum* is present in a biodegraded oil reservoir from Nigeria [62] and a high-temperature sulfur-rich offshore Terra Nova oilfield from Canada [63], suggesting that *Ca. Methanoliparum* thrives in different settings, including acidic and alkaline conditions. *Ca. Methanoliparum* inhabited various types of samples, such as oil refinery sludge [64] and oil sands tailing [65], suggesting that this group of archaea could be potentially applied for in situ bioremediation of oil-contaminated sites.

Our analysis classifies *Ca. Methanoliparum* into four distinct taxa *Ca. Methanoliparum thermophilum*, *Ca. Methanoliparum widdelii*, *Ca. Methanoliparum whitmanii*, and *Ca. Methanoliparum zhangii* (Fig. S1 and Table S11), which is consistent with the previous study [26]. Three of these taxa contain both ACR and MCR complexes, whereas *Ca. Methanoliparum whitmanii* lacks the genes that encode the ACR complex (Table S12). *Ca. Methanoliparum thermophilum* was the dominant cluster according to archaeal 16S rRNA gene amplicon sequencing and metatranscriptomic sequencing in the current study (Fig. S5). This was consistent with previous identification of *Ca. Methanoliparum thermophilum* in hydrocarbon-rich environments [15, 25]*. Ca. Methanoliparum thermophilum*, *Ca. Methanoliparum widdelii*, and *Ca. Methanoliparum zhangii* were abundant, and active in oily sludge samples. The oily sludge samples originating from oil reservoirs is a complex mixture with a high content of hydrocarbons and would represent a special habitat for alkylotrophic methanogens [30, 66]. All four *Ca. Methanoliparum *taxa were active in the SL oilfields. Variations in the abundance and activity of *Ca. Methanoliparum* among oil reservoirs might be due to the oil properties, and geological complexity [67]. This study expanded our view of the diversity of *Ca. Methanoliparum*, and provided insights into their distribution patterns. Factors, such as variations in environmental conditions, and alteration in substrate availability in oil reservoirs can influence the composition of the microbial community [68, 69]. Knowledge regarding suitable growth factors of alkylotrophic methanogens is required.

### Significant role of *Ca. Methanoliparum* in carbon biogeochemical cycle

We compared the activity of *Ca. Methanoliparum*, and hydrocarbon-degrading bacteria both at genome level, and gene level (Tables S7 and S8). Our findings revealed that relative abundance of *acrA*/*Ca. Methanoliparum* increased with degradation degrees (Fig. S2), suggesting alkylotrophic methanogens play a vital role in anaerobic hydrocarbon degradation in subsurface oil reservoirs. *Smithellaceae* were the most active hydrocarbon-degrading bacteria in oil reservoirs, showing higher expression in samples with biodegradation degrees between 0 (TRM, XN), 1 (CQ, YC, ZY), 2 (FS, TRM), 3 (DQ, JH), and 4 (DQ, LH, XJ) (Fig. S3). This result is consistent with previous studies that showed *Smithellaceae* could degrade alkanes via addition to fumarate in syntrophic association with methanogens during the initial degradation period [68, 70–72]. BuS5, the most-studied anaerobic degrader for hydrocarbons [17], is active in the LH oilfield. JS1, widely detected in oil reservoirs [73], is active in the SL oilfield.

Ridge regression model [60] was employed to quantify the relationships between archaea *acrA*/bacteria *assA* + *assA*-like+*bssA* + *nmsA* and degradation degrees on a nonlinear scale (Fig. S6). A simple regression model has formed: Y = 0.01X_1_ + 0.86X_2_ + 0.49. From the fitted equation, we can see the coefficient of X_2_ is larger than X_1_, which means that *Ca. Methanoliparum* played a more significant role in hydrocarbon degradation than bacteria. Overall, these findings suggested that we need to re-evaluate the contribution of alkylotrophic methanogens on the anaerobic hydrocarbon oxidation compared to bacterial systems. Quantification of the contribution in various oil reservoirs needs more evidence from future isotopic fractionation studies [74]. Investigating these microbes further will also provide a better understanding of the role of microorganisms in the fate of petroleum in oil reservoirs deep underground.

**Figure 5:**
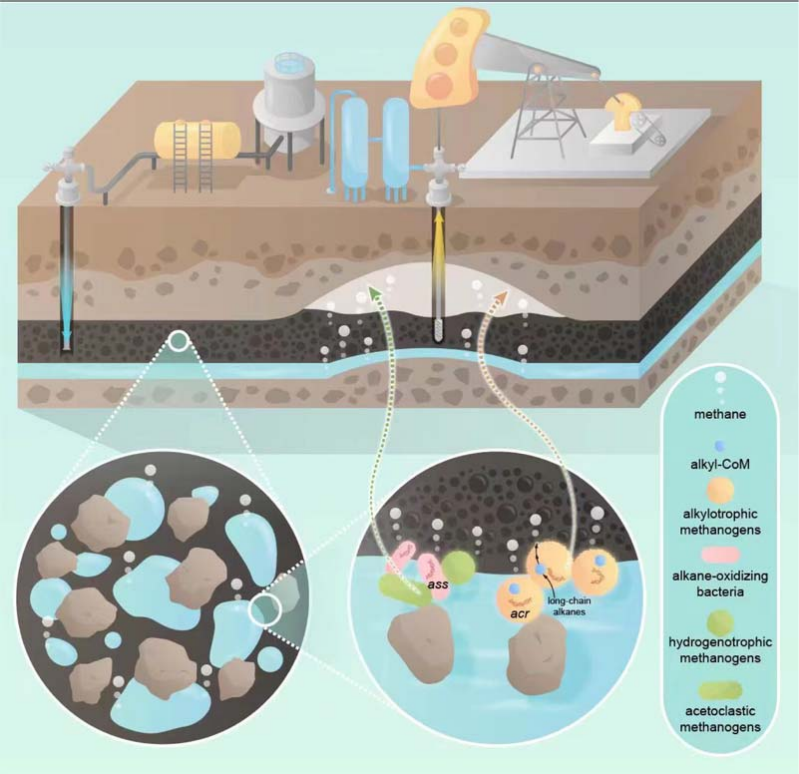
A schematic diagram showing biodegradation mechanisms in subsurface oil reservoirs. The biodegradation process of crude oil occurs at the “crude oil-rock-water” interface. Different degradation models of petroleum hydrocarbons to methane may affect carbon fractionation.

Common enhanced oil recovery (EOR) methods include water flooding, chemical flooding, and microbial flooding [75]. After extraction by these traditional methods, the water content in oil wells will increase significantly in the middle, and late stages of oil reservoirs [76], and these long-established oilfields are facing the challenge of decreased oil production, leaving nearly two-thirds of the oil in the oilfield [77]. Therefore, methanogenic hydrocarbon degradation was proposed as a microbial enhanced energy recovery (MEER) technology that can convert the oil to methane, especially from the exhausted or depleted reservoirs [11, 78]. MEER is environmentally and economically suitable in a reservoir setting [79]. Although the microbial communities from different oil reservoirs vary greatly, the wide distribution, and activity of *Ca. Methanoliparum* in oilfields underscore the pivotal role of alkylotrophic methanogens in subsurface oil reservoirs in China, suggesting that alkylotrophic methanogens have potential for application in MEER (Fig. 5). In MEER, *Ca. Methanoliparum* in the reservoir can be utilized to increase energy recovery by converting difficult-to-recover liquid crude oil into gaseous natural gas. Furthermore, alkylotrophic methanogens are widely present in oil reservoirs, which may disrupt the previously used methods for estimating biogenic methane reserves in reservoirs [80]. The quantification of conversion efficiency and rate under natural hydrocarbon-rich environments require further investigation.

### Collection and storage of oil-related samples and their effects on *Ca. Methanoliparum*

Oil, oily sludge, oil-produced water, and hydrocarbon source rock were the most common sampling types within oilfield systems [77]. Ideally, crude oil and nucleic acid should be extracted immediately after sample collection. However, this is not feasible in many studies, and samples may be stored for a long time after collection. For example, obtaining instantaneous samples from reservoirs is often very challenging [81], as few projects can afford the expensive drilling costs. Microbial oil degradation is a slow process, and in reservoirs may occur on geological time scales[1]. In contrast, storing the samples in an oxygen-limited and low-temperature environment for several years will have a small impact on oil degradation and oil quality. Nevertheless, the effects of sample storage conditions, including oxygen content and storage temperature, on microbial abundance and community are of concern to researchers. First, samples may be affected by oxygen after collection. However, our samples were sealed in the anaerobic bags. Considering the nature of oil sludge samples, oxygen cannot fully penetrate the interior, thus avoiding oxygen stress on internal microbes [4]. In our previous research, after 25 months of storage in anaerobic bags or anaerobic buckets, *Ca. Methanoliparum* could still be successfully enriched [26]. Second, many studies evaluated the effectiveness of refrigeration or freezing on the microbial community and indicated that, compared to −80°C samples, refrigeration at 4°C showed a little alteration in microbial composition [82, 83]. In this research, 8 samples stored at −80°C and 165 samples stored at 4°C were used to test the presence of *Ca. Methanoliparum* based on different molecular analysis methods (Table S13). We found that *Ca. Methanoliparum* could be detected in 87.5% (7/8) of samples stored at −80°C and 84.8% (140/165) of samples stored at 4°C. In addition, based on our long-term enrichment of *Ca. Methanoliparum* at 25°C–75°C, the optimum temperature for *Ca. Methanoliparum* ranged from 35°C to 55°C [26], and the abundance of *Ca. Methanoliparum* would decrease under 4°C storage. In a word, our results from the current study and previous research revealed that *Ca. Methanoliparum* was present in oil-related samples stored for a few months, indicating it is a widely distributed oil degrader in the original oil reservoir.

In summary, we have provided evidence for in situ anaerobic hydrocarbon biodegradation by alkylotrophic methanogens in various oilfields across China. Our result also represents the analysis of *Ca. Methanoliparum* associated with biodegradation degrees, oil reservoirs, and sample types, suggesting that alkylotrophic methanogens preferentially dwell on moderate or severe biodegraded oily sludge samples in most oil reservoirs of China, particularly in the CQ, JS, and SL oilfields. This discovery advances our understanding of alkylotrophic methanogenesis and its role in methane formation in reservoirs, with implications for the energy recovery from large exploited subsurface oil reservoirs.

## Supplementary Material

SupplementaryFigures202404234

MLPSupplementaryTables20240711

## Data Availability

The 16S rRNA gene amplicon sequences, metagenomic, and metatranscriptomic data, and metabolite runs generated in current study are available in the NODE database (https://www.biosino.org/node/project/detail/OEP003873).

## Code availability

The sources of the code, and programs used for analyses are mentioned in the Methods, and are also available at GitHub (https://github.com/cui-jing/Methanoliparum-in-oilfields).
