# Correction to: Anaerobic hydrocarbon biodegradation by alkylotrophic methanogens in deep oil reservoirs

**DOI:** 10.1093/ismejo/wrae196

**Published:** 2024-10-11

This is a correction to: Cui-Jing Zhang, Zhuo Zhou, Guihong Cha, Ling Li, Lin Fu, Lai-Yan Liu, Lu Yang, Gunter Wegener, Lei Cheng, Meng Li, Anaerobic hydrocarbon biodegradation by alkylotrophic methanogens in deep oil reservoirs, *The ISME Journal*, Volume 18, Issue 1, January 2024, wrae152, https://doi.org/10.1093/ismejo/wrae152

The grant number 323250002 has been corrected to read 32325002.

