# Supplementary material for: Anaerobic hydrocarbon biodegradation by alkylotrophic methanogens in deep oil reservoirs
Source: ISME J. 2024 Jul 31;18(1):wrae152. doi: 10.1093/ismejo/wrae152 (PMC11376074; doi:10.1093/ismejo/wrae152)
Supplement: SupplementaryFigures202404234 [file supplementaryfigures202404234.pdf]

Supplementary Figures

Title

Anaerobic hydrocarbon biodegradation by alkylotrophic methanogens in deep oil reservoirs

Authors

Cui-Jing Zhang[#1,3], Zhuo Zhou[#2], Guihong Cha[#2], Ling Li[2], Lin Fu[2], Lai-Yan Liu[2], Lu Yang[2], Gunter Wegener[4,5], Lei Cheng[*2], Meng Li[*1,3]

Affiliations

1, Archaeal Biology Center, Institute for Advanced Study, Shenzhen University, Shenzhen, China.

2, Key Laboratory of Development and Application of Rural Renewable Energy, Biogas Institute of Ministry of Agriculture and Rural Affairs, Chengdu, China.

3, Shenzhen Key Laboratory of Marine Microbiome Engineering, Institute for Advanced Study, Shenzhen University, Shenzhen, China.

4, MARUM, Center for Marine Environmental Sciences, University of Bremen, Bremen, Germany.

5, Max Planck Institute for Marine Microbiology, Bremen, Germany.

#Contributed equally to this work

Notes: Present address for Lu Yang, College of Resources and Environment, Chengdu University of Information Technology, Chengdu, 610225, China

*Corresponding authors

Email:

Meng Li, limeng848@szu.edu.cn

Lei Cheng, chenglei@caas.cn

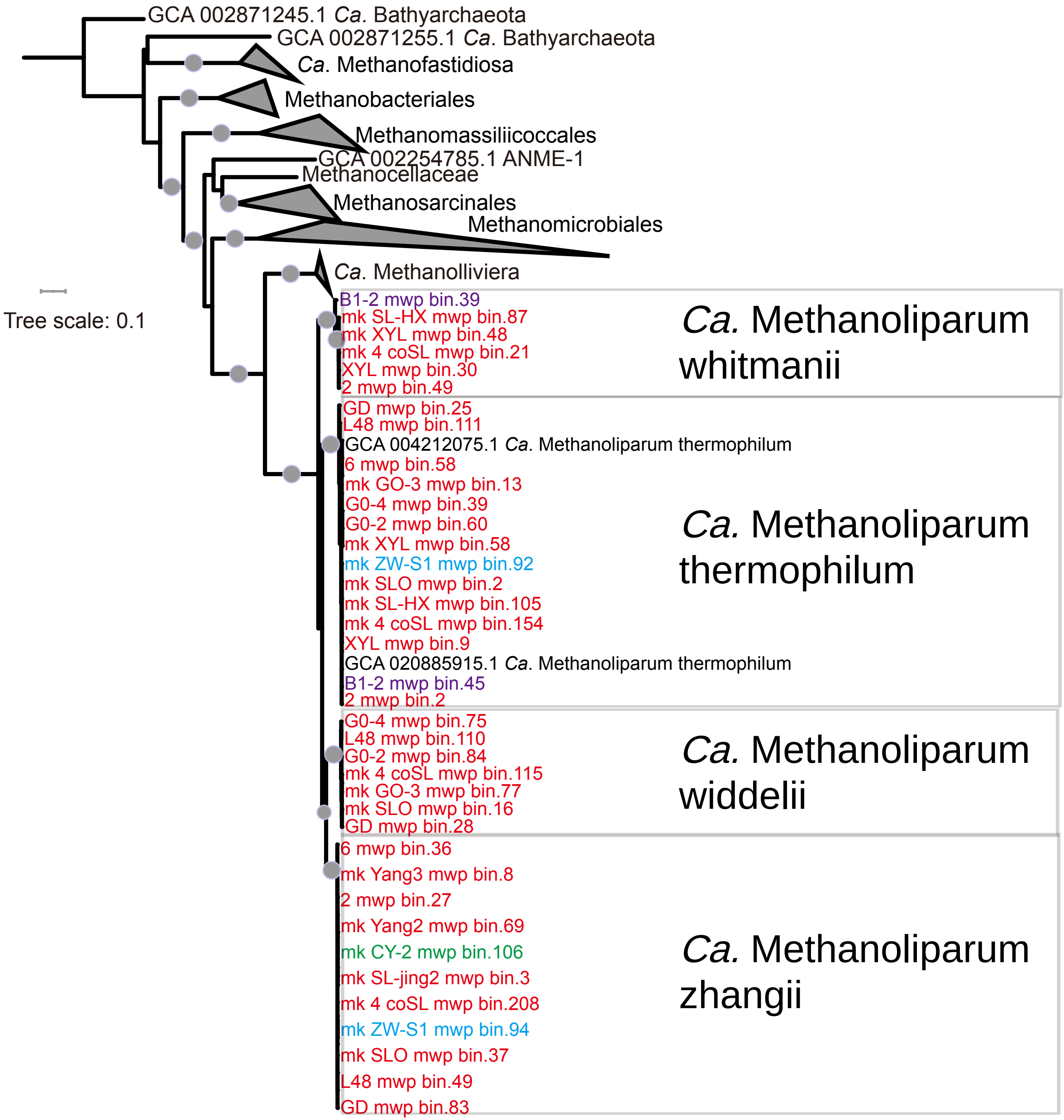

Fig. S1. Phylogeny of *Ca.* Methanoliparum and related methanogens. Phylogenetic tree based on the concatenated alignments of 16 ribosomal proteins by using IQ-TREE with the parameters ' -m WAG, -bb 1000' , root to Ca. Bathyarchaeota. ' Ca. Methanoliparum' MAGs obtained from the CQ, JS, SL, and XJ are indicated by purple, blue, red, and green, respectively.

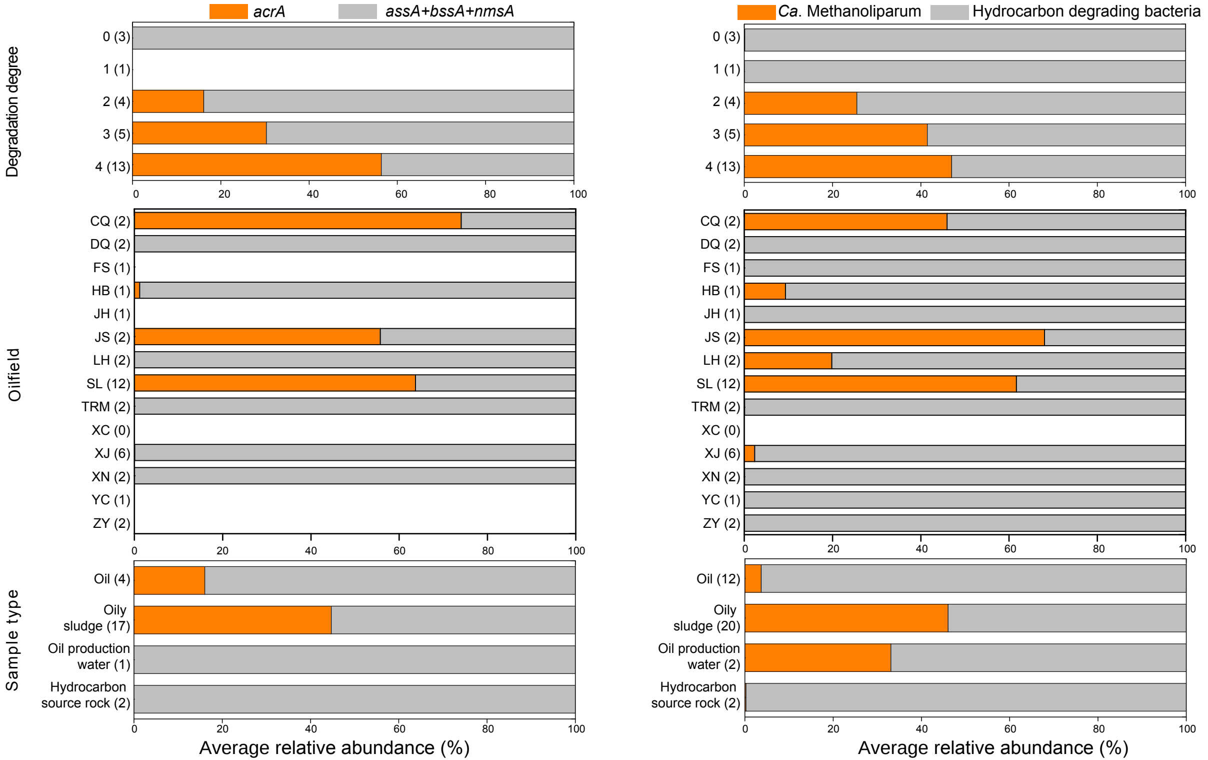

Fig S2. Average relative abundance of *acrA* and *assA+bssA+nmsA* /*Ca*. Methanoliparum and hydrocarbon-degrading bacteria in samples across different degradation degrees, different oil reservoirs and different sample types based on 36 metatranscriptomic datasets. The total number of samples is marked in brackets. Hydrocarbon degrading bacteria are highlighted in Table S7.

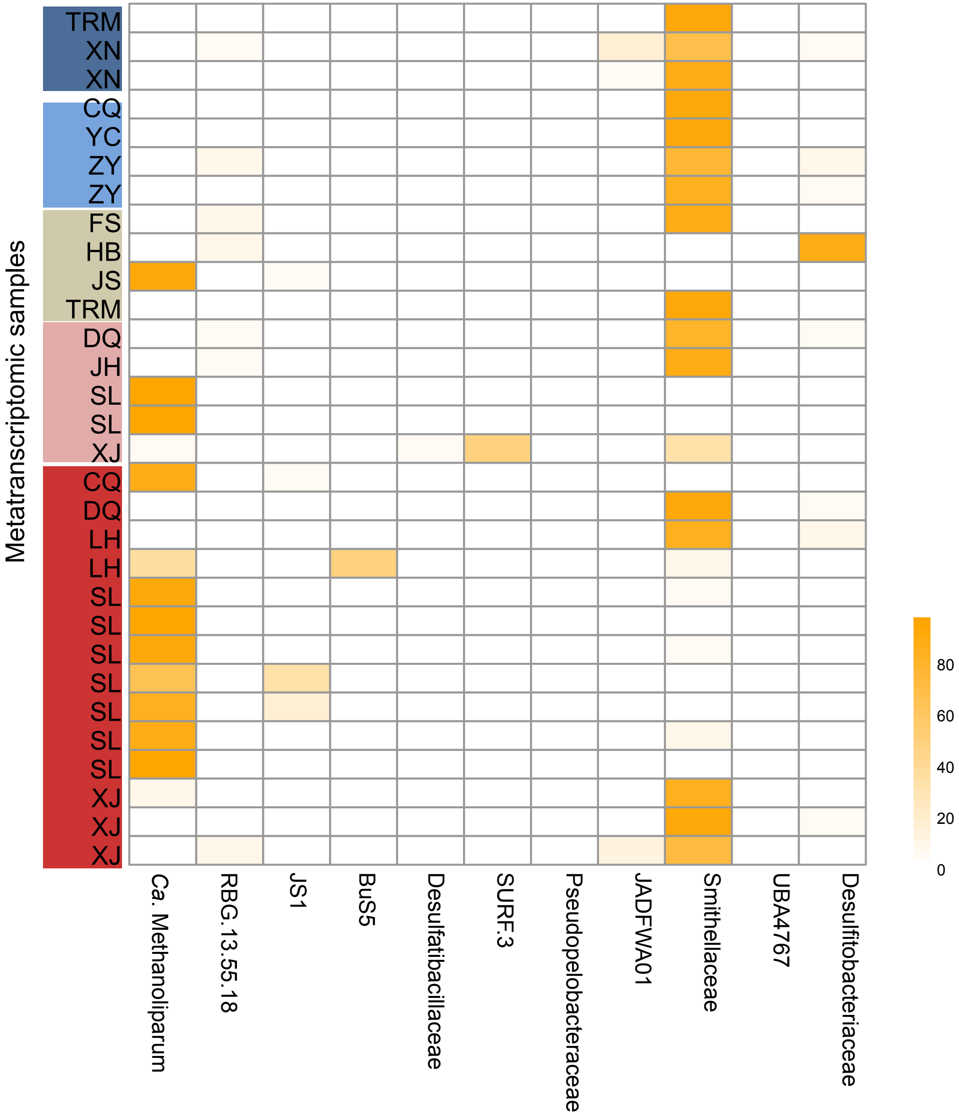

Fig. S3 | Comparison of MAGs abundance for hydrocarbon activation. Relative abundance of *Ca*. Methanoliparum and hydrocarbon-degrading bacteria MAGs in samples across different degradation degrees and different oilfield based on 36 metatranscriptomic data.

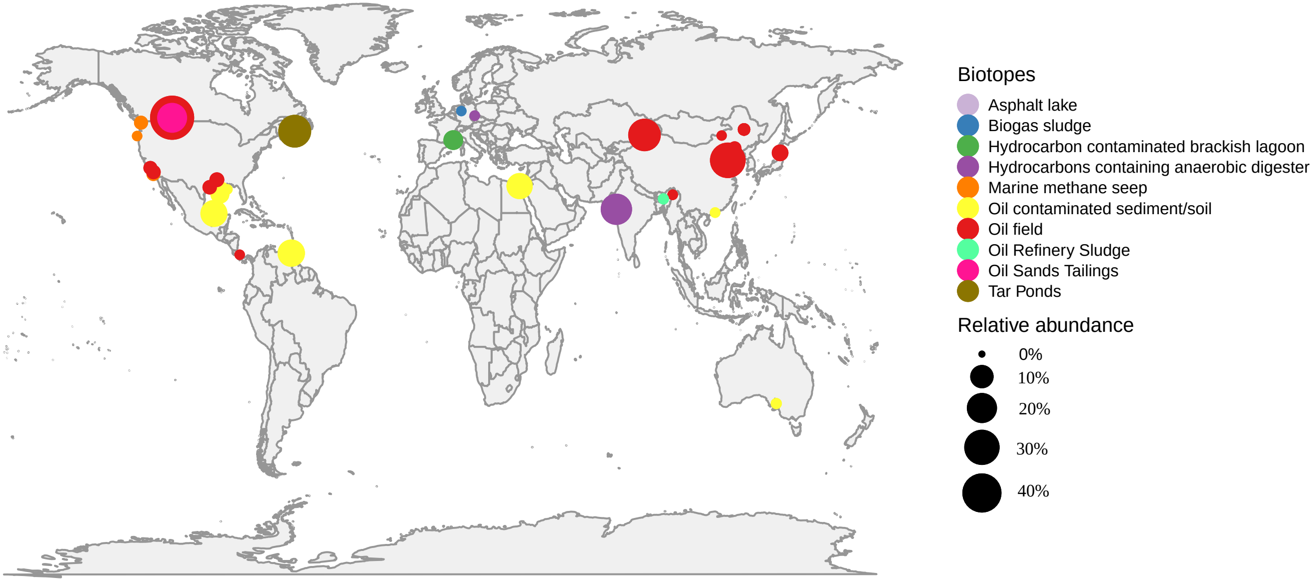

Fig S4. Global distribution of *Ca.* Methanoliparum in crude oil-related biotopes. Node color indicates the type of biotopes, and node size represents the relative abundance. The abundance of 16S rRNA gene sequences of *Ca.* Methanoliparium is relative to total 16S rRNA or archaeal 16S gene sequences according to the primer used in that study (Table S11).

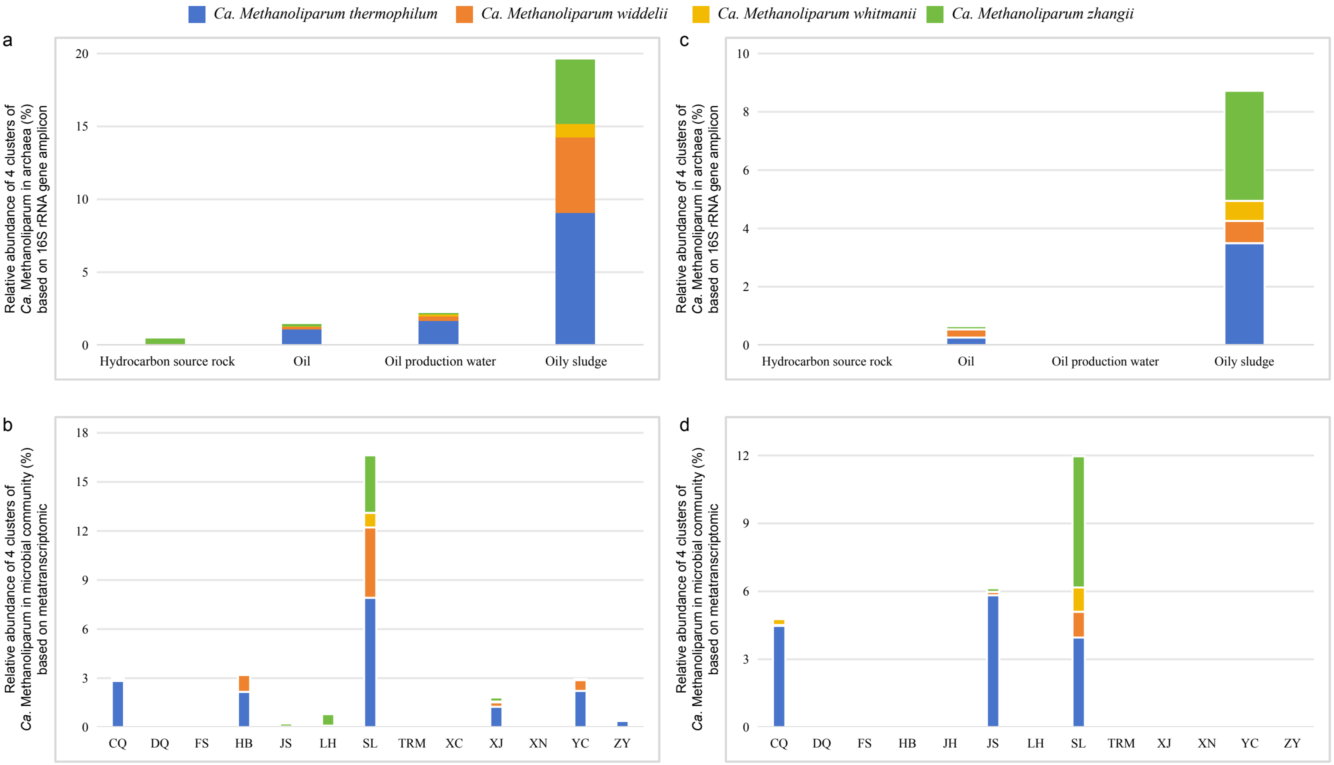

Fig S5. Abundance and activity of 4 clusters of *Ca.* Methanoliparum. Relative abundance of 4 clusters of *Ca.* Methanoliparum in all archaea based on 16S rRNAgene amplicon sequencing across different sample types (**a**) and different oil reservoirs (**b**). Relative abundance of 4 clusters of *Ca.* Methanoliparum in all organisms based on mapping of the metatranscriptomes on the entire 1,346 dereplicated MAGs (Table S7) across different sample typs (**c**) and different oil reservoirs (**d**) .

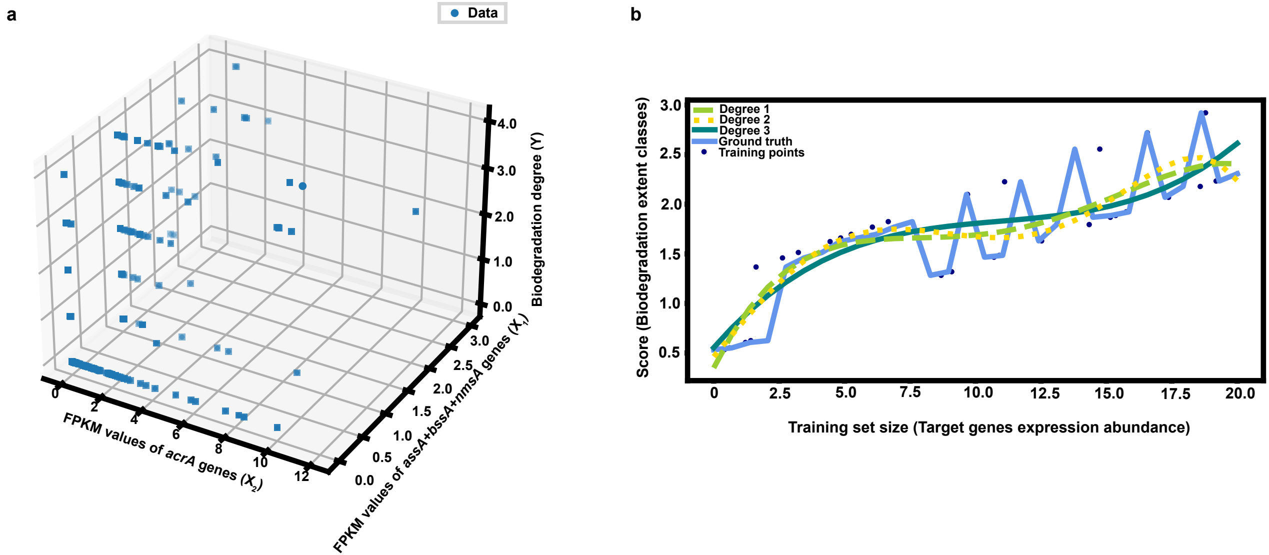

Fig S6. Correlation analysis of differential gene (*acrA, assA, bssA*, and *nmsA*) expression and degradation degrees using ridge regression analysis. Select three eigenvalues to draw a three-dimensional scatterplot. X1-non-*acrA* (bacteria: *assA, bssA*, and *nmsA*) genes expression abundance; X2-*acrA* gene expression abundance; Y-degradation degrees.
